# Multi-Biomarker Profiling and Recurrent Hospitalizations in Heart Failure

**DOI:** 10.3389/fcvm.2016.00037

**Published:** 2016-10-10

**Authors:** Antoni Bayes-Genis, Julio Núñez, Eduardo Núñez, Jaume Barallat Martínez, Maria-Cruz Pastor Ferrer, Marta de Antonio, Elisabet Zamora, Juan Sanchis, Josep Lupón Rosés

**Affiliations:** ^1^Cardiology Service and Heart Failure Unit, Hospital Universitari Germans Trias i Pujol, Badalona, Spain; ^2^Department of Medicine, Autonomous University of Barcelona, Barcelona, Spain; ^3^Cardiology Service, Hospital Clínico Universitario, Valencia, Spain; ^4^University of Valencia, Valencia, Spain; ^5^Biochemistry Service, Hospital Universitari Germans Trias i Pujol, Badalona, Spain

**Keywords:** biomarkers, heart failure, hospitalizations, prediction, NT-proBNP, hs-TnT, ST2

## Abstract

**Background:**

Despite advances in pharmacologic therapy and devices, patients with heart failure (HF) continue to have significant rehospitalization rates and risk prediction remains challenging. We sought to explore the value of a multi-biomarker panel [including NT-proBNP, high-sensitivity cardiac troponin T (hs-TnT), and ST2] on top of clinical assessment for long-term prediction of recurrent hospitalizations in HF.

**Methods and results:**

NT-proBNP, hs-TnT, and ST2 (suppression of tumorigenicity-2) levels were measured in 891 consecutive ambulatory HF patients. The independent association between the multi-biomarker panel and recurrent hospitalizations was assessed through a multivariable negative binomial regression and expressed as incidence rates ratios. McFadden pseudo-*R*^2^ and goodness-of-fit measures were also used. The total number of unplanned hospitalizations [all-cause, cardiovascular (CV)-, and HF-related] were selected as the primary endpoints. At a mean follow-up of 4.2 ± 2.1 years, 1623 all-cause hospitalizations in 498 patients (55.9%), 710 CV-related hospitalizations in 331 patients (37.2%), and 444 HF-related hospitalizations in 214 patients (24.1%) were registered. The crude incidence of all-cause, CV-, and HF-related recurrent hospitalizations was significantly higher for patients with the multi-biomarker panel above the cut-point (hs-TnT > 14 ng/L, NT-proBNP > 1000 ng/L, and ST2 > 35 ng/mL) (all *P* < 0.001). For all-cause, CV-, and HF-related recurrent hospitalizations, the McFadden *R*^2^, Akaike information criterion, and Bayesian information criterion supported the superiority of incorporating the multi-biomarker panel into a clinical predictive model.

**Conclusion:**

A multi-biomarker approach based on NT-proBNP, hs-TnT, and ST2 better identifies HF patients at risk for recurrent hospitalizations as compared to approaches entailing just one or two of these biomarkers. Elucidation of new biophysiological predictors for recurrent hospitalizations may identify patient profiles for focused intervention.

## Introduction

Despite current treatment with evidence-based drugs and devices, patients with heart failure (HF) are frequently admitted to the hospital because of symptom exacerbation, and once admitted, they are often readmitted. HF is the most common cause of hospitalization in patients over age 65, accounting for a total estimated cost of $30.7 billion according to 2016 heart disease and stroke statistics (1); about two thirds of these costs are attributable to HF-related hospitalizations. In patients hospitalized for HF, rates of rehospitalization remain as high as 50% within 6 months of discharge (2). In ambulatory patients, after diagnosis of HF is confirmed, 83% patients are hospitalized at least once and 43% are hospitalized at least four times (3). The perspective of pay-per-performance acts as a catalyst for action to limit recurrent HF hospitalizations.

Risk prediction of recurrent hospitalizations has often been downplayed, with study endpoints focused on time-to-first event analysis (4, 5) and disregarding the impact of recurrent readmissions that frequently occur in HF. Recent initiatives advocate for including recurrent hospitalizations for risk stratification (6, 7) to convey a more realistic and comprehensive picture of the HF continuum. Concerns regarding the need to reduce recurrent hospitalizations must focus on the prediction of which patients with HF are more likely to be readmitted. Risk stratification may be refined by the use of biomarkers for different pathobiological pathways that established clinical risk factors fail to unmask.

NT-proBNP is indicative of neurohormonal activation and myocardial strain (8), high-sensitivity cardiac troponin T (hs-TnT) is a surrogate of myocyte injury (9), and ST2 reflects myocardial fibrosis, remodeling, and inflammation (10). These markers, either alone or in combination, are independently associated in time-to-first event analyses with outcomes in patients with HF (11–13). However, the value of a multi-biomarker profile to refine the risk of recurrent hospitalizations in HF is ill defined. Accordingly, we sought to examine the value of a multi-biomarker panel including NT-proBNP, hs-TnT, and ST2 on top of clinical assessment for long-term prediction of recurrent hospitalizations in HF. The state-of-the-art statistics used, including multivariable negative binomial regression (NBreg) models and incidence rates ratios (IRRs), foregrounds the impact of recurrent hospitalizations.

## Materials and Methods

### Study Population

From May 2006 to July 2010, ambulatory patients treated at a multidisciplinary HF clinic were consecutively included in the study in an outpatient setting. Referral inclusion criteria and blood sample collection have been described elsewhere (11, 14). In summary, patients were referred to the HF clinic by cardiology or internal medicine departments and, to a lesser extent, from the emergency or other hospital departments. The principal referral criterion was HF according to the European Society of Cardiology guidelines irrespective of etiology, at least one hospitalization for HF, or a reduced left ventricular ejection fraction (LVEF). NT-proBNP, hs-TnT, and ST2 were analyzed from the same blood sample stored at −80°, without previous freeze–thaw cycles. All samples were obtained between 09:00 a.m. and 12:00 p.m.

All participants provided written informed consent, and the local ethics committee approved the study. All study procedures were in accord with the ethical standards outlined in the Helsinki Declaration of 1975, as revised in 1983.

### Follow-up and Outcomes

All patients were followed at regular predefined intervals, with additional visits as required in case of decompensation. The regular visitation schedule included a minimum of quarterly visits with nurses, biannual visits with physicians, and elective visits with geriatricians, psychiatrists, and rehabilitation physicians (11, 14). Patients who did not attend the regular visits were contacted by telephone.

The total number of unplanned hospitalizations [all-cause, cardiovascular (CV)-, and HF-related] were selected as the primary endpoints. CV admissions were considered as those occurring due to acute coronary syndrome, arrhythmias, stroke, or other CV causes such as rupture of an aneurysm, peripheral ischemia, or aortic dissection. Hospitalizations were identified from clinic records of patients in the HF unit and hospital wards and from the electronic Catalan history record. Fatal events were identified from the clinical records from the HF unit, hospital wards, emergency room, and general practitioners, and by contacting the patient’s relatives. Furthermore, data were verified from the databases of the Catalan and Spanish Health Systems. Six patients lost during follow-up were adequately censored.

### NT-proBNP Assay

NT-proBNP levels were determined using an immuno-electrochemiluminescence method (Elecsys^®^, Roche Diagnostics, Switzerland). This assay has <0.001% cross-reactivity with bioactive BNP, and in the constituent studies in this report, the assay had inter-run coefficients of variation ranging from 0.9 to 5.5%.

### High-Sensitivity Cardiac Troponin T Assay

Troponin levels were measured by electrochemiluminescence immunoassay using the hs-TnT assay on the Modular Analytics E 170 (Roche Diagnostics). The hs-TnT assay has an analytic range from 3 to 10,000 ng/L. At the 99th percentile value of 13 ng/L, the coefficient of variation was 9%.

### ST2 Assay

ST2 was measured from plasma samples using a high-sensitivity sandwich monoclonal immunoassay (Presage^®^ ST2 assay, Critical Diagnostics, San Diego, CA, USA). The ST2 assay had a within-run coefficient of <2.5%, a total coefficient of variation of 4%, and a limit of detection of 1.31 ng/mL.

### Statistical Analysis

Continuous variables were expressed as mean ± 1 SD or median [interquartile range (IQR)] when appropriate. Normal distribution was assessed by normal *Q*–*Q* plots. Discrete variables were summarized as percentages. Clinical characteristics were compared among patients with none, one, two, or three or more HF-related hospitalizations and also among biomarker strata. *P* values for trend for continuous variables and for each HF etiology were calculated using Spearman correlation. Categorical variables were compared using chi-square test (linear by linear).

Biomarker strata relied on categorical use of the three biomarkers using widely accepted cut-points, namely, 1000 ng/L for NT-proBNP, 14 ng/L for hs-TnT, and 35 ng/mL for ST2. Four strata, each indicative of the number of biomarkers elevated (0, 1, 2, and 3) were defined. Crude incidence rates (expressed as number of readmissions per 100 person-years) were calculated for each biomarker strata and for each readmission endpoint (all-cause, CV-, and HF-related). Also event-free curves from Cox regression analysis were plotted for the first and second CV-related hospital admission relative to biomarker strata.

The independent association between biomarkers and recurrent hospitalizations was assessed through a multivariable NBreg and expressed as IRR. The category “0” of the variable (none of the biomarkers elevated) was used as reference.

In addition to the three studied biomarkers, the covariates included in the clinical models were as follows for each endpoint: (1) all-cause recurrent hospitalizations – age, NYHA class, time since HF diagnosis, diabetes mellitus, peripheral artery disease, chronic obstructive pulmonary disease, glomerular filtration rate, serum sodium, hemoglobin, and treatment with beta-blockers; (2) CV-recurrent hospitalizations – age, NYHA class, time since HF diagnosis, diabetes mellitus, peripheral artery disease, glomerular filtration rate, serum sodium, treatment with beta-blockers, ACEI/ARB, and loop diuretics; and (3) HF-related recurrent hospitalizations – age, gender, NYHA class, time since HF diagnosis, diabetes mellitus, glomerular filtration rate, sodium, treatment with beta-blockers, and loop diuretics. The decision to include a covariate in each model was mainly based on backward stepwise selection with Akaike information criterion (AIC) as stopping criterion (*P* = 0.16) to achieve a parsimonious model and prevent model overfitting. During this process, the linearity assumption for continuous variables was simultaneously tested, and transformed if appropriate, with fractional polynomials (15).

The McFadden pseudo-*R*^2^ and measures of goodness-of-fit such as the AIC and the Bayesian information criterion (BIC) were used to compare the performance of each multi-biomarker model vs. the clinical model without biomarkers. Given any two estimated models, the model with the lower BIC and AIC scores was preferred. No statistical tests compare different BIC or AIC estimations, and lower values indicate a better model. These comparisons were made, in turn, for each recurrent hospitalization.

A two-sided *P* value of <0.05 was considered to be statistically significant for all analyses. All analyses were performed using Stata 14.1 (StataCorp. 2015, Stata Statistical Software: Release 14.1 College Station, TX, USA: StataCorp LP) and SPSS 15 (SPSS Inc., Chicago, IL, USA).

## Results

### Patients

From May 2006 to July 2010, a total of 891 consecutive HF patients were included in this analysis. Table 1 shows the baseline characteristics of the study cohort relative to the occurrence of none, one, two, and three or more HF-related hospitalizations. The mean age of the total cohort was 66.4 ± 12.4 years, 71.6% were male, 52.7% showed ischemic etiology, and 87.5% had LVEF below 50%. Most of them were in NYHA class II (65.5%). The median concentrations (IQR) for NT-proBNP, hs-TnT, and ST2 were 1237 ng/mL (481–2798), 22.4 ng/mL (10.5–40.2), and 38.1 ng/mL (30.8–50.9), respectively. The number of HF hospitalizations tended to increase with age, HF duration, worse NYHA functional class, the presence of diabetes mellitus, renal dysfunction, and anemia, and higher biomarker concentrations.

**Table 1:** **Demographic and clinical characteristics relative to the number of HF-related hospitalizations**.

	Number of HF-related hospitalizations				*P* value
	None (*n* = 677)	One (*n* = 103)	Two (*n* = 62)	Three or more (*n* = 49)	
Age, years	65.2 ± 12.8	71.3 ± 10	70.1 ± 9.9	68.6 ± 9.4	<0.001
Males	501 (74.0)	70 (68.0)	40 (64.5)	27 (55.1)	0.001
White	671 (99.1)	103 (100)	62 (100)	49 (100)	0.25
Etiology					
Ischemic heart disease	354 (52.3)	64 (62.1)	31 (50.0)	21 (42.9)	0.89
Dilated cardiomyopathy	64 (9.5)	10 (9.7)	7 (11.3)	5 (10.2)	0.70
Hypertensive	57 (8.4)	8 (7.8)	8 (12.9)	9 (18.4)	0.08
Alcohol	40 (5.9)	3 (2.9)	3 (4.8)	4 (8.2)	0.62
Toxic (drugs)	20 (3.0)	1 (1.0)	1 (1.6)	1 (2.0)	0.24
Valvular	103 (11.2)	12 (11.7)	10 (16.1)	7 (14.3)	0.31
Other	66 (9.7)	5 (4.9)	2 (3.2)	2 (4.1)	0.01
Heart failure duration, months	25.6 (3.7–67.8)	29.2 (11–96)	36 (9.5–92.8)	36 (8.6–95.6)	0.003
LVEF, %	32.8 ± 12.2	34.9 ± 15.6	35.9 ± 16.3	33.9 ± 16.3	0.40
NYHA functional class III–IV	157 (23.2)	42 (40.7)	25 (40.3)	18 (36.7)	<0.001
Hypertension	407 (60.1)	67 (65.0)	42 (67.7)	35 (71.4)	<0.05
Diabetes mellitus	218 (32.2)	39 (37.9)	32 (51.6)	25 (51.0)	<0.001
BMI, kg/m^2^	26.8 (24–30.4)	26.4 (23.9–31.1)	27.4 (25.5–30.2)	28.3 (25.8–31.8)	0.04
eGFR, mL/min/1.73 m^2^	56.8 ± 26.9	43.4 ± 24.4	45.7 ± 18.9	43.5 ± 27.6	<0.001
Sodium, mmol/L	139 ± 3.4	139.3 ± 3.5	139 ± 3.2	139.5 ± 3.7	0.14
Hemoglobin, g/dL	13 ± 1.9	12.2 ± 1.9	12.6 ± 1.5	12.5 ± 1.9	<0.001
Treatments, *n* (%)					
ACEI or ARB	630 (93.1)	82 (79.6)	57 (91.9)	45 (91.81)	0.13
Beta-blocker	625 (92.3)	90 (87.4)	51 (82.3)	42 (85.7)	0.004
Spironolactone/eplerenone	368 (54.4)	59 (57.3)	49 (79.0)	37 (75.5)	<0.001
Loop diuretic	606 (89.5)	103 (100)	62 (100)	49 (100)	<0.001
Digoxin	258 (38.1)	47 (45.6)	40 (64.5)	33 (67.3)	<0.001
NT-proBNP, ng/L	1008 (388–2495)	1867 (744–4907)	1721 (938–3512)	1847 (843–3682)	<0.001
ST2, ng/mL	37.1 (30.3–49.1)	40.5 (33–53)	44.41 (31.1–65.5)	41.1 (32–58)	0.001
Hs-TnT, ng/L	19 (8.6–36.9)	32.6 (19–52.2)	28.1 (19.1–44.1)	32.5 (19.4–48.8)	<0.001

*Data are mean ± SD, median (IQR), or *n* (%)*.

*ACEI, angiotensin-converting enzyme inhibitor; ARB, angiotensin II receptor blocker; BMI, body mass index; eGRF, estimated glomerular renal filtration (CKD-EPI equation); hs-TnT, high-sensitivity troponin T; LVEF, left ventricular ejection fraction; NYHA, New York Heart Association; NT-proBNP, N-terminal pro-brain natriuretic peptide; ST2, soluble ST2*.

Table 2 summarizes patient characteristics relative to the multi-biomarker strata of none elevated or one, two, or three biomarkers above the cut-point. As the number of biomarkers above the cut-point increased, patients tended to be older, with shorter HF duration, worse NYHA functional class, and worse biochemistry parameters (estimated glomerular filtration rate, sodium, hemoglobin) (Table 2).

**Table 2:** **Differences among patients based on the number of biomarkers above the cut-point**.

	Number of biomarkers above the cut-point				*P* value
	None (*n* = 129)	One (*n* = 210)	Two (*n* = 256)	Three (*n* = 296)	
Age, years	58.7 ± 10.8	62.2 ± 12.8	68.4 ± 11.2	71.1 ± 11.2	<0.001
Males	89 (69.0)	154 (73.3)	179 (69.9)	216 (73.0)	0.61
White	127 (98.4)	209 (99.5)	254 (99.2)	295 (99.7)	0.40
Etiology					
Ischemic heart disease	63 (48.8)	105 (50.0)	131 (51.2)	171 (57.8)	0.04
Dilated cardiomyopathy	20 (15.5)	24 (11.4)	23 (9.0)	19 (6.4)	0.003
Hypertensive	13 (10.1)	15 (7.1)	27 (10.5)	27 (9.1)	0.82
Alcohol	11 (8.5)	19 (9.0)	14 (5.5)	6 (2.0)	<0.001
Toxic (drugs)	2 (1.6)	8 (3.8)	6 (2.3)	7 (2.4)	0.84
Valvular	6 (4.7)	22 (10.5)	29 (11.3)	48 (16.2)	0.001
Other	14 (10.9)	17 (8.1)	26 (10.2)	18 (6.1)	0.14
Heart failure duration, months	42.2 (16.1–75)	36 (10.6–75.1)	25.4 (3–80.1)	21 (2–61)	<0.001
LVEF, %	33.4 ± 11.7	33.8 ± 13.5	33.3 ± 14.2	33.1 ± 12.8	0.53
NYHA functional class III–IV	14 (10.9)	31 (14.8)	74 (28.9)	123 (41.6)	<0.001
Hypertension	67 (51.9)	109 (51.9)	168 (65.6)	207 (69.9)	<0.001
Diabetes mellitus	31 (24.0)	64 (30.5)	99 (38.7)	120 (40.5)	<0.001
BMI, kg/m^2^	27.7 (25.6–31.6)	28.1 (25–31.4)	26.7 (24.6–30.6)	25.6 (22.9–28.9)	<0.001
eGFR, mL/min/1.73 m^2^	70.1 ± 21.6	62.5 ± 27.3	50.2 ± 24.5	43.3 ± 23.3	<0.001
Sodium, mmol/L	140.2 ± 2.5	139.4 ± 3.3	139.5 ± 3.3	138.4 ± 3.8	<0.001
Hemoglobin, g/dL	13.5 ± 1.6	13.4 ± 1.8	12.8 ± 1.8	12.3 ± 1.8	<0.001
Treatments, *n* (%)					
ACEI or ARB	129 (100)	201 (95.7)	239 (93.4)	245 (82.8)	<0.001
Beta-blocker	124 (96.1)	200 (95.2)	224 (87.5)	260 (87.8)	<0.001
Spironolactone/eplerenone	58 (45.0)	115 (54.8)	157 (61.3)	183 (61.8)	0.001
Loop diuretic	109 (84.5)	191 (91.0)	242 (94.5)	277 (93.6)	0.002
Digoxin	36 (27.9)	85 (40.5)	115 (44.9)	142 (48.0)	<0.001
NT-proBNP, ng/L	281 (138–543)	549 (230–835)	1403 (755–2472)	3190 (1852–3190)	<0.001
ST2, ng/mL	27.8 (24.3–31.1)	34.5 (29.5–42.2)	35 (30.4–45.6)	51.7 (42.7–70.7)	<0.001
Hs-TnT, ng/L	7.3 (4–9.6)	11.5 (6.5–20.9)	26.4 (16.1–40.9)	38.1 (26.9–60.5)	<0.001

*Data are mean ± SD, median (IQR), or *n* (%)*.

*ACEI, angiotensin-converting enzyme inhibitor; ARB, angiotensin II receptor blocker; BMI, body mass index; eGRF, estimated glomerular renal filtration (CKD-EPI equation); hs-TnT, high-sensitivity troponin T; LVEF, left ventricular ejection fraction; NYHA, New York Heart Association; NT-proBNP, N-terminal pro-brain natriuretic peptide; ST2, soluble ST2*.

### Outcomes

At a mean follow-up of 4.2 ± 2.1 years, 399 (44.8%) deaths, 1623 all-cause hospitalizations in 498 patients (55.9%), 710 CV-related hospitalizations in 331 patients (37.2%), and 444 HF-related hospitalizations in 214 patients (24.1%) were registered. The distribution of patients with one, two, three, four, and five or more all-cause, CV-, and HF-related hospitalizations is depicted in Figure S1 in Supplementary Material. Crude incidences for all-cause, CV-, and HF-related recurrent hospitalizations were 36, 19, and 12 per 100 person-years, respectively.

### Multi-Biomarker Profiling and Recurrent Hospitalizations

**Figure 1:**
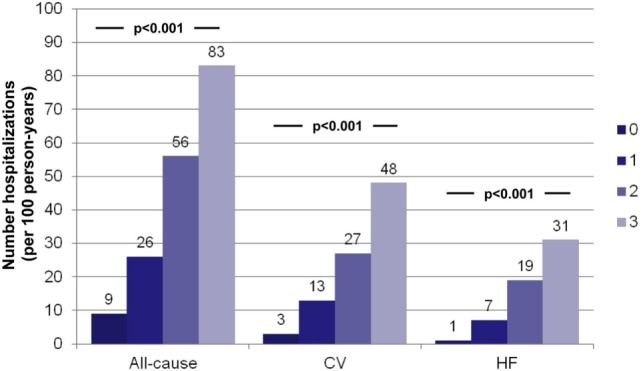
**Crude incidences (per 100 person-years) of hospitalizations relative to the number of elevated biomarkers above the predefined cut-points**. Crude incidence of all-cause, CV-related, and HF-related hospitalizations was very significantly related to the number of elevated biomarkers. hs-TnT > 14 ng/L, NTproBNP > 1000 ng/L, and ST2 > 35 ng/mL.

For patients with the multi-biomarker panel above the cut-point (hs-TnT > 14 ng/L, NT-proBNP > 1000 ng/L, and ST2 > 35 ng/mL), the crude incidences of all-cause, CV-, and HF-related recurrent hospitalizations were significantly higher (all *P* < 0.001; Table 3). Relative to the number of elevated biomarkers, a stepwise increase in the incidences of all-cause and specific causes of recurrent hospitalizations was found when moving from none to three biomarkers elevated (all *P* < 0.001; Figure 1). Indeed, the number of HF-related hospitalizations per 100 person-years significantly rose from 1 to 31 from none to three biomarkers elevated (*P* < 0.001).

**Table 3:** **Hospitalization crude incidence rates (per 100 person-years) relative to biomarker levels**.

Biomarker	Above cut-point	Below cut-point	*P* value
**All-cause hospitalizations**			
hs-TnT	52	16	<0.001
NT-proBNP	52	23	<0.001
ST2	47	24	<0.001
**CV-related hospitalizations**			
hs-TnT	28	7	<0.001
NT-proBNP	30	10	<0.001
ST2	25	13	<0.001
**HF-related hospitalizations**			
hs-TnT	18	4	<0.001
NT-proBNP	19	6	<0.001
ST2	16	8	<0.001

*Cut-points: hs-TnT, 14 ng/L; NT-proBNP, 1000 ng/L; ST2, 35 ng/mL*.

**Figure 2:**
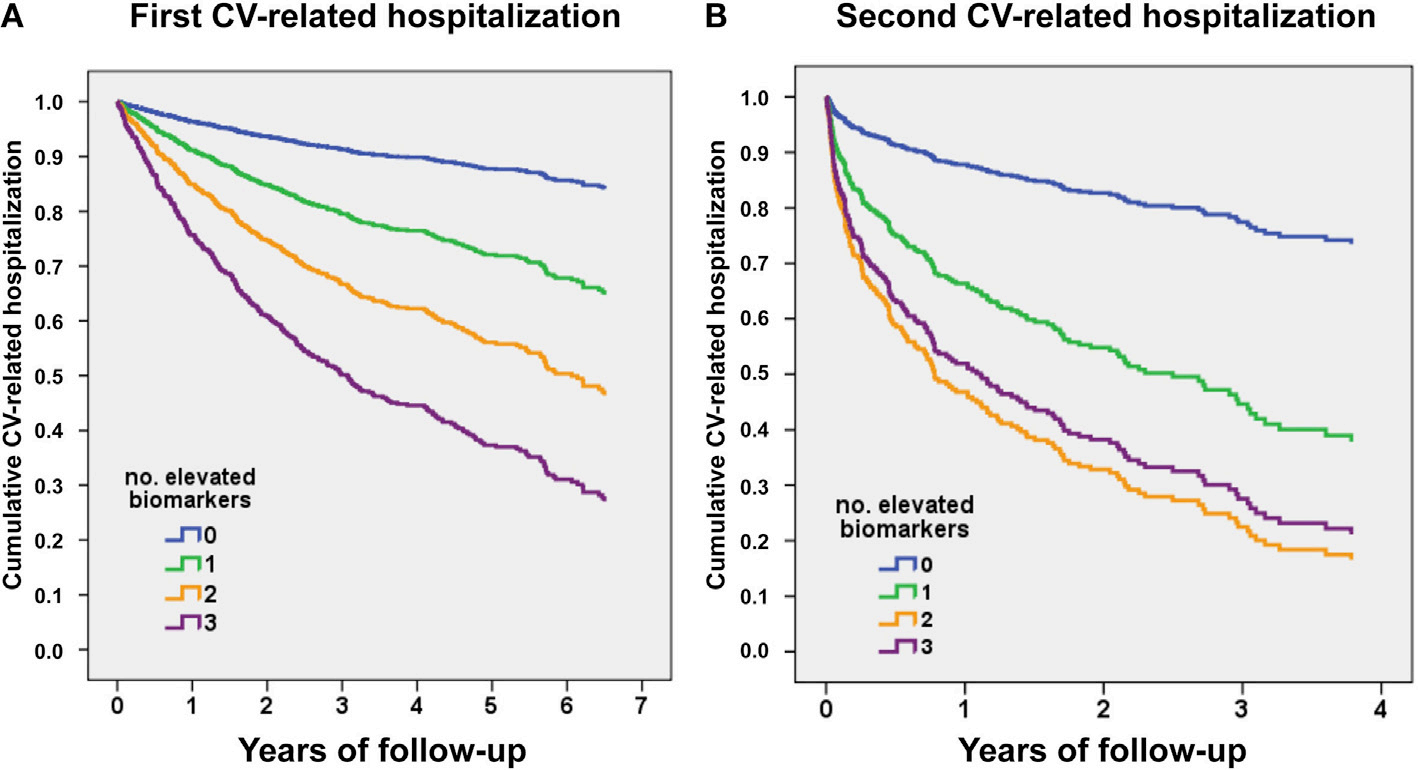
**Event-free survival curves for CV-related recurrent hospitalizations, relative to the number of elevated biomarkers above the predefined cut-points**. **(A)** First CV-related hospitalization. **(B)** Second CV-related hospitalization (from discharge date of the first CV-related hospitalization). hs-TnT > 14 ng/L, NTproBNP > 1000 ng/L, and ST2 > 35 ng/mL.

Figure 2A shows event-free curves for first CV-related admission. Hazard ratios (HRs) based on the number of biomarkers elevated (taking 0 as reference) were as follows: one elevated biomarker, HR 2.57 (95% CI 1.52–4.34), *P* < 0.001; two elevated biomarkers, HR 4.54 (95% CI 4.74–7.54), *P* < 0.001; and three elevated biomarkers, HR 7.29 (95% CI 4.4–12.1), *P* < 0.001. Figure 2B shows event-free curves for the second CV-related admission, considering from discharge date of the first CV-related hospitalization. HRs based on the number of biomarkers elevated (taking 0 as reference) were as follows: one elevated biomarker, HR 3.42 (95% CI 1.03–11.4), *P* < 0.05; two elevated biomarkers, HR 5.58 (95% CI 1.75–17.9), *P* = 0.004; and three elevated biomarkers, HR 5.16 (95% CI 1.62–16.4), *P* = 0.006.

**Figure 3:**
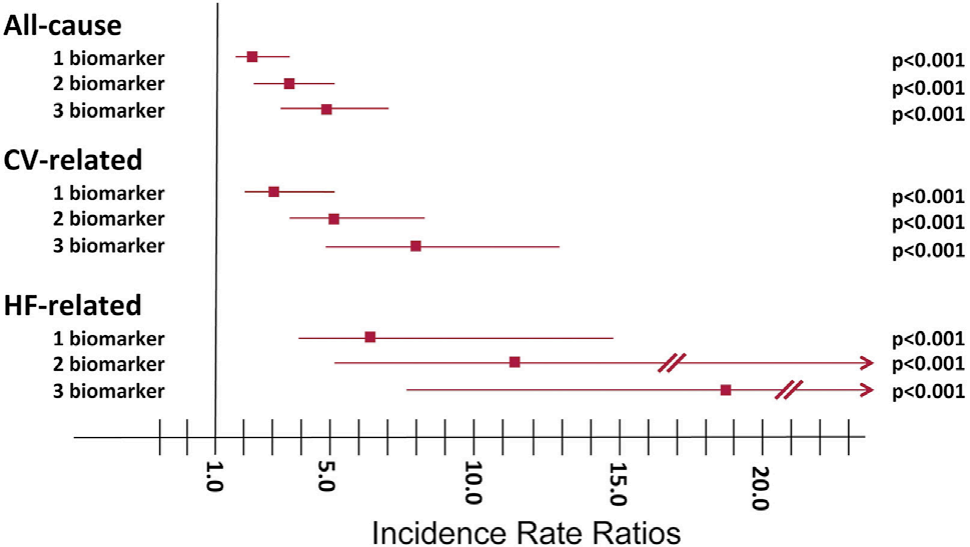
**Incidence rate ratios (IRRs) of recurrent hospitalizations relative to the number of elevated biomarkers above the predefined cut-points**. All-cause hospitalizations: one elevated biomarker, IRR 2.36 (95% CI 1.62–3.42), *P* < 0.001; two elevated biomarkers, IRR 3.64 (95% CI 2.52–5.26), *P* < 0.001; three elevated biomarkers, IRR 4.87 (95% CI03.38–7.03), *P* < 0.001. CV-related hospitalizations: one elevated biomarker, IRR 3.16 (95% CI 1.94–5.15), *P* < 0.001; two elevated biomarkers, IRR 5.11 (95% CI 3.17–8.25), *P* < 0.001; three elevated biomarkers, IRR 7.94 (95% CI 4.87–12.95), *P* < 0.001. HF-related hospitalizations: one elevated biomarker, IRR 6.15 (95% CI 2.55–14.80), *P* < 0.001; two elevated biomarkers, IRR 11.37 (95% CI 4.82–26.82), *P* < 0.001; three elevated biomarkers, IRR 18.80 (95% CI 7.88–44.84), *P* < 0.001.

After multivariable adjustment, gradients of risk remained significant for all endpoints, with the strongest association for HF-related hospitalizations. Figure 3 shows the corresponding IRRs (95% CIs) for 1, 2, or 3 biomarkers elevated compared to none for all-cause, CV-, and HF-related recurrent hospitalizations.

For all-cause, CV-, and HF-related recurrent hospitalizations, the McFadden *R*^2^ as well as the goodness-of-fit measures AIC and BIC supported the value of incorporating the multi-biomarker panel with NT-proBNP, hs-TnT, and ST2 into a clinical predictive model (Table 4).

**Table 4:** **Model performance measures**.

	Clinical model	Multi-biomarker model	Difference
**All-cause readmission**			
Model’s log-likelihood	−1657.01	−1611.83	45.18
McFadden *R*^2^	0.08	0.11	0.03
AIC	3338.02	3253.66	84.36
BIC	3395.53	3325.55	69.98^a^
**CV-related readmissions**			
Model’s log-likelihood	−1247.12	−1208.16	38.96
McFadden *R*^2^	0.08	0.11	0.03
AIC	2518.24	2446.32	71.92
BIC	2575.75	2518.21	57.54^a^
**HF-related readmission**			
Model’s log-likelihood	−852.02	−819.34	32.68
McFadden *R*^2^	0.09	0.12	0.04
AIC	1726.04	1666.68	59.36
BIC	1778.76	1733.78	44.98^a^

*Multi-biomarker model – NT-proBNP, hs-TnT, and ST2 on top of clinical model*.

*Clinical model – see Section “Materials and Methods” for details*.

*^a^Differences >10 are defined as very strong in favor of the model with lower values*.

Finally, in a sensitivity analysis, we tested the interaction between a multimarker risk score (TnThs + NTproBNP + ST-2) and eGFR < 60 mL/min/1.73 m^2^ under the same multivariate scenario. This analysis showed a non-significant interaction among multimarker score and eGFR, revealing, a non-differential prognostic effect of this proposed score across renal function strata (omnibus *P*-value for all-cause and CV-readmission were 0.381 and 0.112, respectively). Importantly, the multimarker risk score was strongly associated with the risk of HF readmissions in patients with and without renal dysfunction; however, the magnitude of the association was greater in patient without renal dysfunction.

## Discussion

Most HF readmissions reflect a rise in cardiac filling pressures, yet clinical symptoms have not been sufficiently reliable to detect early decompensation or responsive enough to allow timely reintervention to avoid hospitalization (16). Thus, levels of cardiac biomarkers have the potential to anticipate risk of recurrent hospitalization. Our study shows that multi-biomarker profiling with NT-proBNP, hs-TnT, and ST2 permits prediction of recurrent hospitalizations above and beyond clinical risk factors.

In 2008, Braunwald (17) classified circulating biomarkers into categories based on their pathophysiological effects in HF and postulated that multiple biomarkers in combination would provide a valuable means of risk stratification. A number of multi-biomarker combinations to predict outcomes in chronic HF have since been tested. The Penn HF Study included high-sensitivity C-reactive protein, uric acid and myeloperoxidase, B-type natriuretic peptide, soluble fms-like tyrosine kinase receptor-1, troponin I, and ST2 (18). Patients in the highest multi-biomarker tertile had a nearly 14-fold unadjusted risk of death, transplant, or ventricular assist device placement compared to the lowest tertile. Next, Lupón et al. investigated NT-proBNP, ST2, and hs-TnT to determine the relative role of each in all-cause mortality (14). Again, all three biomarkers incorporated into a clinical model yielded better measures of performance at 1-, 2-, and 3-year follow-up. These two studies explored the addition of a multi-biomarker panel to predict mortality in addition to a clinical score but did not explore admissions or recurrent hospitalizations.

A recent report characterized measurements of NT-proBNP, ST2, GDF-15, and hs-TnT in the cohort from the PROTECT (ProBNP Outpatient Tailored Chronic Heart Failure) study (19), which accounted for HF admissions as time-to-first event but not recurrent hospitalizations. By contrast, in our study, all-cause, CV-related, and HF-related recurrent hospitalizations were examined. All-cause–related and CV-related recurrent hospitalizations were 9-fold and 16-fold higher (in terms of HF-related hospitalizations per 100 person-years), respectively, if the three biomarkers were elevated. Regarding HF-related recurrent hospitalizations, after a mean 4.2 years of follow-up, the number of these hospitalizations per 100 person-years was only one if all of the biomarkers were below the cut-point and 31 if the three studied biomarkers were elevated.

Identifying the “optimal” panel of biomarkers for assessing HF remains a formidable task, given the predictive value of the studied biomarkers in the different studies. A large prospective multicenter study which includes all the biomarkers to identify the best panel is warranted. In the present study, among the multiple biomarkers for HF, natriuretic peptides (neurohormonal activation and ventricular strain), troponins (myocyte injury), and ST2 (fibrosis, ventricular remodeling, and inflammation) were chosen because they are currently available, US Food and Drug Administration (FDA) approved, and already acknowledged in the American College of Cardiology/American Heart Association guidelines because of their strong prognostic potential in chronic HF (20). The cut-points used in this study were either approved by the FDA (i.e., 35 ng/mL for ST2) (21), identified by a multitude of clinical reports (i.e., 1000 ng/L for NT-proBNP) (22, 23), or the upper reference limit (99th percentile) was provided by the manufacturer (i.e., 14 ng/L for hs-TnT).^1^

It remains uncertain whether biomarkers can be harnessed as a tool to guide HF therapy and subsequently reduce recurrent hospitalizations. Biomarkers have potential for therapy guidance with ACE inhibitors, ARBs, MRAs, β blockers, and diuretics. However, at present, insufficient data exist to support therapy titration to target lower NT-proBNP, hs-TnT, and ST2 levels. Only natriuretic peptides have been prospectively tested, with conflicting results, in part due to disparities in trial designs. The Guiding Evidence-Based Therapy Using Biomarker Intensified Treatment in Heart Failure (GUIDE-IT) study is designed to definitively assess the effects of a natriuretic peptide–guided strategy in patients with systolic HF on clinically relevant endpoints of mortality, hospitalization, quality of life, and medical resource use (24). Our data support the need for further research aiming to evaluate the clinical effect of multi-biomarker-guided strategies.

The limitations of the use of time-to-first event statistical approaches for recurrent events are obvious. In chronic diseases such as HF, time-to-first event ignores the prognostic impact of recurrent hospitalizations, thus providing an incomplete epidemiological picture of the disease. As demonstrated by Rogers et al. (25), the inclusion of recurrent events provides a considerable gain in statistical power compared with the time-to-first event approach. Yet, the most appropriate statistical method for analysis of recurrent events remains controversial (25). The approach we followed – an NBreg – not only exploits the Poisson distributional assumption of recurrent events but also accounts for interdependence between recurrent hospitalizations; a further adjustment for potential heterogeneity was handled by estimating robust SEs. It is unfortunate, however, that the traditional discrimination and reclassification indices available in time-to-first event analysis have not been developed yet for recurrent event statistics.

Some limitations must be acknowledged with regard to the present study. First, whether serial measurements of three biomarkers at predefined time points would have improved risk stratification was not incorporated into the design and is beyond the scope of the present report (26). Second, the population was a general HF population treated at a specific and multidisciplinary HF unit in a tertiary care hospital; the criteria for deciding patient admission were homogeneous and according to institutional guidelines. Third, our cohort was composed mainly of patients with HF of ischemic etiology and with reduced ejection fraction. As such, these results cannot be extrapolated to a global HF population.

## Conclusion

Our findings support the value of a multi-biomarker approach that incorporates NT-proBNP, hs-TnT, and ST2 to predict the risk of recurrent hospitalizations for worsening HF as well as for CV-related and all causes. Elucidation of new biophysiological predictors for recurrent hospitalizations may be most useful to reveal patient profiles for focused intervention.

## Author Contributions

AB-G: draft of the manuscript, study design, data analysis, and review of the final version of the manuscript. JN, EN, JB, PC, MA, EZ, JS, and JR: study design, data analysis, and review of the final version of the manuscript.

## Conflict of Interest Statement

The authors declare that the research was conducted in the absence of any commercial or financial relationships that could be construed as a potential conflict of interest.

## Footnotes

^1^Available from: http://www.canada.com/content/dam/roche_canada/en_ca/documents/package_inserts/troponint-hs-05092744190-english-v6-can.pdf
