# Supplementary material for: Multi-Biomarker Profiling and Recurrent Hospitalizations in Heart Failure
Source: Front Cardiovasc Med. 2016 Oct 10;3:37. doi: 10.3389/fcvm.2016.00037 (PMC5056426; doi:10.3389/fcvm.2016.00037)
Supplement: Supplementary file 1 [file Image_1.PDF]

Suppl Figure 1

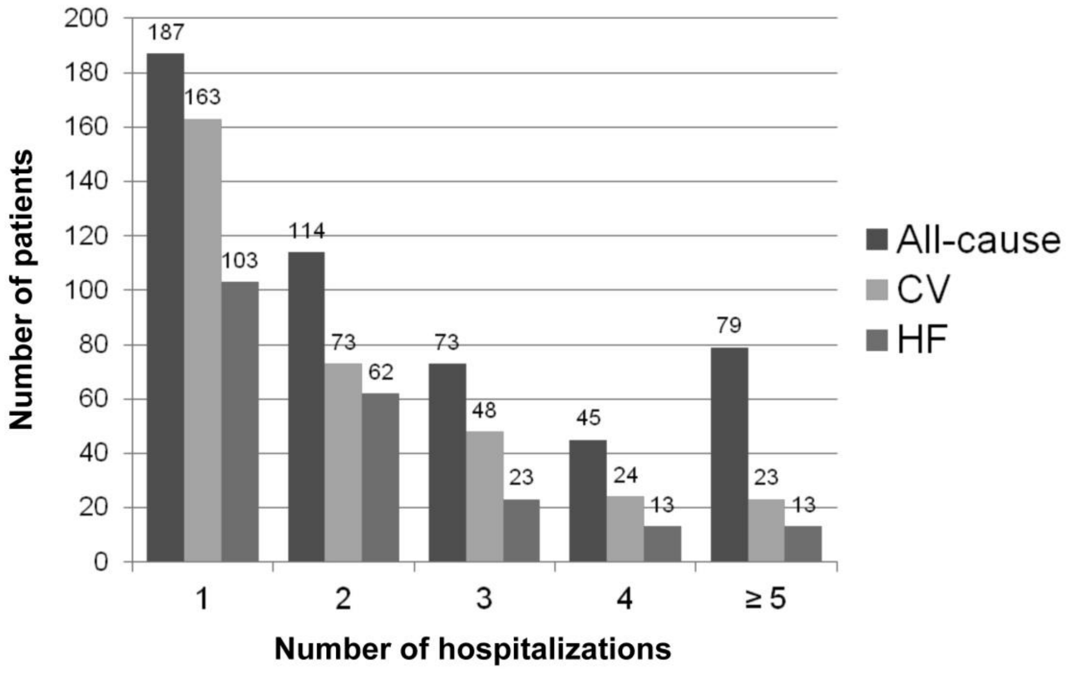

Supplementary Figure 1. **Distribution of patients with one, two, three, four, and five or more hospitalizations.**
